# Comments on "The role of serum adropin in determining the clinical outcomes of patients with traumatic brain injury: a case-control study"

**DOI:** 10.1590/1806-9282.20240999

**Published:** 2024-10-07

**Authors:** Şebnem Zeynep Eke Kurt

**Affiliations:** 1 University of Health Sciences, Taksim Training and Research Hospital, Department of Emergency Medicine – İstanbul, Turkey.

Dear Editor,

I read with great interest the article by Tataroğlu et al. entitled "The role of serum adropin in determining the clinical outcomes of patients with traumatic brain injury: a case-control study"^1^. This study provides valuable insights into the potential use of adropin as a biomarker in TBI. However, we believe that several aspects of the study warrant further discussion and clarification.

First, while the authors demonstrate a significant increase in adropin levels in TBI patients compared to controls, the clinical implications of this finding remain unclear. The study shows higher adropin levels in patients with better outcomes (those discharged from the emergency department), but it does not establish a clear prognostic value for adropin in TBI. A more detailed analysis of the relationship between adropin levels and specific clinical outcomes would strengthen the study's conclusions. Previous studies have highlighted the importance of establishing a clear prognostic value for biomarkers in TBI^2^.

Second, the authors suggest that adropin levels could be used to monitor the tissue healing process and potential complications after trauma. However, the study only measured adropin levels at a single time point in the acute phase. To substantiate this claim, a longitudinal study design with serial measurements of adropin levels would be required to demonstrate its utility in monitoring recovery and identifying complications. Longitudinal studies have been critical in understanding the dynamics of other TBI biomarkers^3^.

We appreciate that the authors acknowledge the limitations of the study, particularly the lack of follow-up adropin measurements. We agree that long-term studies are needed to determine the time course of adropin levels after injury and their return to baseline. In addition, it would be valuable to investigate how adropin levels correlate with other established biomarkers of TBI and with neuroimaging findings. The combination of multiple biomarkers has been shown to improve prognostic accuracy in TBI^4,5^.

The study touches on the potential protective role of adropin in trauma patients, but the mechanisms remain speculative. Future research should aim to elucidate the pathways involved and explore whether adropin directly influences the healing process or serves as an indirect marker of other protective mechanisms. Recent research has highlighted the neuroprotective effects of several peptides in TBI^6^.

Despite these limitations, this study opens an interesting avenue for research on the role of adropin in TBI. Future studies could explore the molecular mechanisms by which adropin may exert neuroprotective effects in the context of TBI. In addition, investigating the relationship between adropin and other physiological parameters, such as intracranial pressure or cerebral perfusion, may provide additional insight into its potential clinical utility. Studies linking biomarkers to intracranial pressure and cerebral perfusion have provided valuable insights into TBI management^7,8^.

I hope that these comments will contribute to a more complete understanding of the role of adropin in TBI and stimulate further research in this promising area.
